# Intracellular expression of FLT3 in Purkinje cells: implications for adoptive T-cell therapies

**DOI:** 10.1038/s41375-018-0330-7

**Published:** 2019-01-03

**Authors:** Neşe Çakmak-Görür, Josefine Radke, Simone Rhein, Elisa Schumann, Gerald Willimsky, Frank L. Heppner, Thomas Blankenstein, Antonio Pezzutto

**Affiliations:** 1 Max Delbrück Center for Molecular Medicine in Helmholtz Association, Berlin, Germany; 2 Charité-Universitätsmedizin, corporate member of Freie Universität Berlin, Humboldt-Universität zu Berlin, and Berlin Institute of Health, Berlin, Germany; 3 Berlin Institute of Health (BIH), Berlin, Germany; 4 Department of Neuropathology, Charité-Universitätsmedizin Berlin, corporate member of Freie Universität Berlin, Humboldt-Universität zu Berlin, and Berlin Institute of Health, Berlin, Germany; 5 German Cancer Consortium (DKTK), Heidelberg, Germany, Partner Site Berlin, Berlin, Germany; 6 Institute of Immunology, Charité-Universitätsmedizin, corporate member of Freie Universität Berlin, Humboldt-Universität zu Berlin, and Berlin Institute of Health, Berlin, Germany; 7 German Cancer Research Center (DKFZ), Heidelberg, Germany; 8 German Center for Neurodegenerative Diseases (DZNE), Berlin, Germany

**Keywords:** Cancer immunotherapy, Acute myeloid leukaemia

## To the Editor:

Despite new drug development and improved understanding of molecular pathways leading to acute myeloid leukemia (AML), the cure rate of the disease is still poor. For high-risk patients or in relapse, the only long-term curative therapy is still allogeneic hematopoietic stem cell transplantation (allo-HSCT), which is not feasible in a considerable number of patients and poses significant risks in morbidity and mortality. New strategies are urgently needed to improve the cure rate in AML.

The success of chimeric antigen receptor (CAR)-modified T-cells directed to the CD19 antigen for the treatment of lymphoid malignancies of B-cell lineage boosted the research on CAR T-cells targeting myeloid antigens, such as CD33, CD123, and Fsm-like tyrosine kinase 3 (FLT3) [1, 2]. FLT3, a tyrosine kinase receptor thought to be specific to hematopoietic lineage, is an attractive target especially due to overexpression in AML blasts. In the past few years, several independent groups developed CAR T-cells targeting FLT3 with promising in vitro results [3–5]. The expression of FLT3 in HSCs might indeed cause concern [5]. Recent publications have shown CD34^+^-HSC are less susceptible to CAR T-cell treatment compared to AML cells [3, 4]. This is likely due to different affinities of the antibodies used for CAR generation, suggesting different CARs might have different toxicities in vivo.

T-cell receptor (TCR)-modified T-cell therapy is an alternative form of adoptive T-cell therapy. Unlike CARs, which recognize cell surface antigens, TCRs recognize peptides derived from any cellular proteins presented by major histocompatibility complex (MHC) molecules. Therefore, TCRs should not be affected by down modulation of surface antigen expression when intracellular expression is retained, which is an escape mechanism in CAR therapies. We have generated an HLA-A2-restricted, FLT3-specific TCR to target AML cells with the aim of using it in the context of HLA-A2-mismatched allo-HSCT. In the proposed set-up, FLT3-directed CD8^+^ T-cells would kill any remaining HLA-A2^+^/FLT3^+^ cells possibly eradicating all leukemic cells while allowing the engraftment and establishment of an HLA-A2^-^, leukemia free hematopoiesis.

**Fig. 1:**
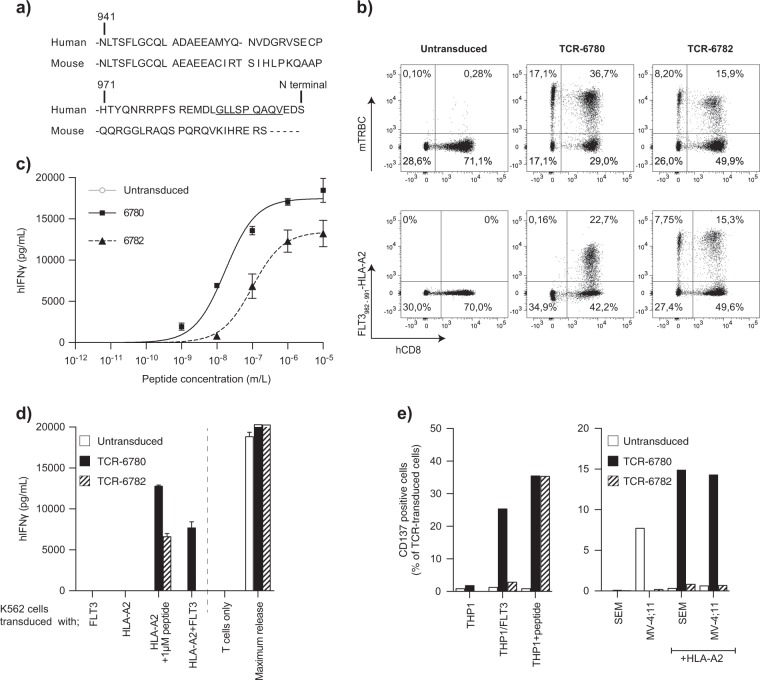
Characterization of FLT3-specific TCRs generated from *ABabDII* mice. **a**) Alignment of human and mouse FLT3 protein spanning the FLT3_982-991_ T-cell epitope GLLSPQAQV used for immunization. Full proteins are 83% identical to each other with N-terminal regions sharing minimum homology. **b**) Flow cytometry analysis of human PBMCs transduced with TCR-6780 and TCR-6782. The cells were stained with antibodies against human CD8, mTRBC, and FLT3_982-991_-HLA-A2 tetramer three days after the transduction. TCR-transduced CD8^+^ T-cells bind to the FLT3_982-991_-HLA-A2 tetramer showing correct pairing of TCR α and β variable chains. The transduction rate varied between 20 and 60% in different experiments depending on the retrovirus titer. **c**) IFN-γ release by TCR-transduced T-cells upon co-culture with T2 cells loaded with increasing concentration of GLL peptide. **d**) Recognition of K562 cells overexpressing HLA-A2 and FLT3 showing natural processing and presentation of FLT3_982-991_ epitope. **e**) CD137 upregulation on the surface of the TCR-transduced CD8^+^ T-cells upon co-culture with cell lines expressing FLT3. THP1 cells (FLT3^+^/HLA-A2^+^) were recognized only by TCR-6780 when FLT3 was overexpressed. SEM and MV-4;11 cell lines (FLT3^+^/HLA-A2^-^) induced CD137 on TCR-6780-transduced T-cells when HLA-A2 was introduced by transduction. FACS plots and graphs represent results from one out of three donors

It is not possible to generate optimal-affinity TCRs against self-antigens in humans because of thymic selection. To circumvent tolerance and develop optimal-affinity TCR against FLT3, we immunized mice expressing a diverse human TCR repertoire and a mouse CD8-binding HLA-A2 molecule (AB*ab*DII) with an in silico-predicted FLT3 epitope [6]. The FLT3_982-991_ peptide GLLSPQAQV (GLL), with an affinity of 56 nM to HLA-A2 predicted by NetMHC 3.4, was selected for immunizations (Fig. 1a). AB*ab*DII mice were immunized and boosted with GLL peptide and peripheral blood was analyzed for peptide-specific T-cells 7 days after immunization. Reactive CD8^+^ T-cells were collected from inguinal lymph nodes and spleen of two mice (#6780 and #6782) and expanded with peptide in vitro for 10 days. By an IFN-γ capture assay, 2,500 and 65,300 IFN-γ^+^-CD8^+^ T-cells were sorted, respectively (Supp. Figure 1). TCRs were isolated by RACE-PCR and Vα and Vβ pairs were identified based on their abundance (Supp. Table 1). We constructed codon-optimized TCR cassettes with mouse constant region for better expression in human T-cells (named TCR-6780 and TCR-6782) in the γ-retroviral vector pMP71 as described [7].

Following retroviral transduction, GLL-specific TCRs were expressed in human CD8^+^ T-cells, as confirmed by staining against the mouse constant TCRβ region (mTRBC) and FLT3_982-991_-HLA-A2 tetramers (Fig. 1b). Transduced T-cells were co-cultured with T2 cells loaded with decreasing concentration of GLL peptide at 1:1 ratio overnight, IFN-γ was detected in the supernatant by ELISA. Both TCR-6780 and TCR-6782 recognized GLL peptide at a concentration as low as 10 nM (Fig. 1c). Half-maximum IFN-γ release at 17 and 100 nM peptide concentrations, respectively, indicates TCR-6780 has a higher functional avidity, although the affinity still appears to be suboptimal.

We generated K562 cell lines expressing HLA-A2, FLT3, or both by γ-retroviral transduction for co-cultivation assays (Supp. Figure 2). TCR-transduced T-cells recognized K562/HLA-A2 cells loaded with the GLL peptide (Fig. 1d). TCR-6780-transduced T-cells secreted IFN-γ to K562 cells co-expressing HLA-A2 and FLT3, proving the epitope was naturally processed and presented while TCR-6782-transduced T-cells did not secrete IFN-γ, indicating a lower functional TCR avidity (Fig. 1d). Next, TCR-6780 and TCR-6782 transduced T-cells were co-cultured with cells expressing FLT3 endogenously (THP1 is derived from AML patient; MV;4-11 is a mixed-lineage myelomonocytic leukemia line carrying the 4;11 chromosomal translocation; SEM is a B-cell precursor leukemia cell line). SEM and MV;4-11 cells were retrovirally transduced to stably express HLA-A2. The target cells were labeled with 1 µM CFSE prior to overnight co-culture with TCR-transduced CD8^+^ T-cells and CD137 upregulation specifically on TCR-transduced CD8^+^ T-cells was analyzed (Supp. Fig 3 for gating strategy). TCR-6780-transduced T-cells recognized cell lines co-expressing FLT3 and HLA-A2 to a different extent. 1.8% of TCR-6780-transduced CD8^+^ T-cells upregulated CD137 in response to THP1 cells in contrast to 25.33% in response to THP-1 overexpressing FLT3. SEM and MV;4-11 were recognized when they expressed HLA-A2 only (14.9% and 14.3%, respectively). TCR-6782-transduced CD8^+^ T-cells did not upregulate CD137, likely because of low avidity (Fig. 1e). We could not detect IFN-γ from effector cells upon co-culture with target cells indicating low avidity of both TCRs (data not shown).

Several FLT3 inhibitors are being used for the treatment of AMLs without life-threatening side effects or toxicity [8]. Anti-FLT3 monoclonal antibodies have entered a clinical trial in AML. TCR-engineered T-cells are more potent in terms of killing target cells. Theoretically, a single molecule of the antigen presented on the MHC can elicit T-cell toxicity [9]. Hence, TCR-mediated on-target recognition of vital tissues may induce severe toxicity [10–12]. So far, there was no evidence in the literature that non-hematopoietic human cells express FLT3 and *Flt3* knock-out mice develop normally but have an impaired lymphoid progenitor cell compartment [13], suggesting FLT3 may indeed be specific to the hematopoietic compartment.

**Fig. 2:**
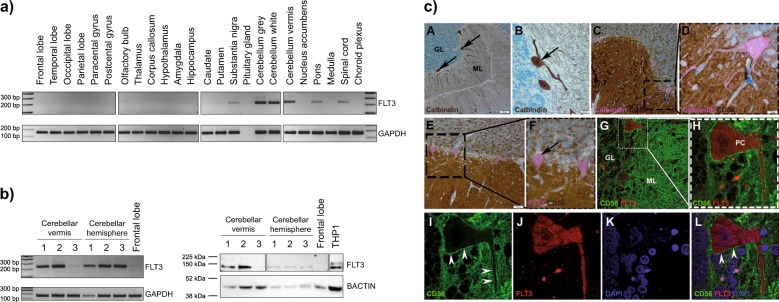
Expression profiling of FLT3 in normal human brain. **a**) FLT3 was amplified from commercially available nerve tissue cDNA array representing 24 different anatomical sites of the normal human brain. High expression was detected in the cerebellum. **b**) RT-PCR (left) and western blot analysis (right) detected FLT3 expression in human cerebellar samples. FLT3 amplicon was detected from cDNA of fresh cerebellar samples from three different healthy donors. For western blot analysis, 100 µg total cerebellar protein extract and 40 µg total protein from the THP1 cell line were separated on SDS-PAGE gel for immunoblotting. While the 160 kDa band corresponding to plasma membrane associated, glycosylated FLT3 was not detected, the 135 kDa band of underglycosylated FLT3 associated with endoplasmic reticulum membrane was detected using total cerebellar protein extract. Protein lysate of the frontal lobe was used as negative control and THP1 cell line served as positive control. **c**) Immunohistochemical analysis of the cerebellum with granular layer (GL), Purkinje cell layer, and molecular layer (ML). The molecular layer contains the dendritic arbors, the Purkinje cell layer the cell bodies of Purkinje neurons (PC) that strongly express calbindin (A, arrows; B, arrow). Double immunohistochemical staining of PCs reveal strong cytoplasmic expression of calbindin (C, D), membranous expression of CD56 (NCAM; C-F) and cytoplasmic expression of FLT3 (E, F, arrow). Confocal microscopy with double immunofluorescence staining shows a maximum projection of a stack of images (G, H) and a single plane of one stack of images at a higher magnification (I–L). CD56 (NCAM, green) is localized on the cell membrane (I, arrowheads) whereas FLT3 (red) is exclusively found in the cytoplasm of PCs (J). No co-localization of CD56 and FLT3 was detected on the cytoplasmic membrane (L, arrowheads)

To assess the safety of FLT3 as a target antigen, we used a cDNA library representing 48 healthy human tissues. We first verified the sensitivity of the PCR reaction on a titration of FLT3^+^ THP1 cell line (Supp. Fig 4a). In RT-PCR, a 236 bp *FLT3* amplicon was detected in several tissues including the brain (Supp. Fig 4b). Testing an additional cDNA library spanning 24 tissues of the central nervous system (CNS) by RT-PCR revealed that *FLT3* was highly expressed in the cerebellar hemispheres and vermis (Fig. 2a). Detected PCR products were confirmed to be *FLT3* by Sanger sequencing (Supp. Fig 4c), thus, unveiling those sites as potential sites of on-target off-tumor toxicity.

Next, we analyzed postmortem human brain tissue. Total protein and total RNA from three different individuals were isolated from the cerebellar vermis (CV) and cerebellar hemispheres (CH). *FLT3* gene expression was verified by RT-PCR (Fig. 2b). FLT3 is synthesized and glycosylated in the endoplasmic reticulum (ER), then transported to the cell membrane. The membrane-bound, glycosylated FLT3 is detected as 160 kDa band while ER-bound immature FLT3 is 135 kDa. We detected only 135 kDa immature FLT3  in the cerebellar lysate by western blot analysis suggesting FLT3 is found primarily intracellular, most likely bound to the ER membrane in cerebellum  tissue (Fig. 2b). In mice, *Flt3* has been detected in the mouse cerebellum, specifically in Purkinje cells (PC) [14]. Immunohistochemical (IHC) and immunofluorescent (IF) staining were used to further elucidate which cerebellar cell type expresses FLT3. Immunofluorescent co-staining of CD56 (neural cell adhesion molecule (NCAM)) and FLT3 revealed a cytoplasmic and intracellular expression of FLT3 in Purkinje cells, confirming the western blot results (Fig. 2c).

In conclusion, we generated two TCRs recognizing the self-epitope FLT3_982-991_ presented in the context of HLA-A2. We showed for the first time that the FLT3_982-991_ epitope is naturally processed and presented and therefore a potential TCR target. The first TCRs we generated did not show optimal-affinity, hence, could not be used for kill assays on AML cell lines as well as on-target toxicity assessment. However, the unexpected intracellular expression of FLT3 in Purkinje cells appears to bear a risk that is too high to move forward with TCR into the clinic because of the anticipated on-target off-tumor recognition. Purkinje cells integrate afferent signals mainly from the brainstem and send their axons down to the deep cerebellar nuclei. PC degeneration causes coordination deficits, such as limb ataxia, dysarthria, and oculomotor disturbances, as seen in paraneoplastic syndromes. Therefore, we abandoned the effort to generate a TCR with improved affinity by novel immunizations and the whole strategy of targeting FLT3 with TCR-transduced T-cells. Nevertheless, our findings do not necessarily infer that FLT3 is not a suitable target for CAR therapy. First, antibody-based therapeutics may not be efficient enough to kill a cell if the surface expression is weak, while few pMHC complexes might be enough for recognition and killing by TCRs [9, 15]. Second and most important, according to our data, FLT3 is likely to be present intracellularly but not on the surface of human Purkinje cells and is therefore unlikely to be recognized by a CAR. The urgent need for novel therapies in AML might well justify pursuing this strategy in high-risk patients.

## Supplementary information

Supplementary materials and methods

Supplementary figure 1

Supplementary figure 2

Supplementary figure 3

Supplementary figure 4

Supplementary table 1
