# Supplementary material for: Intracellular expression of FLT3 in Purkinje cells: implications for adoptive T-cell therapies
Source: Leukemia. 2019 Jan 3;33(4):1039–43. doi: 10.1038/s41375-018-0330-7 (PMC6484709; doi:10.1038/s41375-018-0330-7)
Supplement: Supplementary file 1 — Supplementary materials and methods [file 41375_2018_330_MOESM1_ESM.docx]

### Supplementary Materials and Methods

### Cell lines

The human AML cell line THP1 (FLT3^+^/HLA-A2^+^) was purchased from ATCC. The human B cell precursor leukemia cell line SEM and the human AML cell line MV;4-11 (both FLT3^+^/HLA-A2^-^) were purchased from Leibniz Institute DSMZ-German Collection of Microorganisms and Cell Cultures. THP1 cell line was cultivated in RPMI 1640 medium supplemented with 10% FCS and 50 µM β-mercaptoethanol. Human CML cell line K562 and MV-4;11 were maintained in RPMI medium supplemented with 10% FBS. SEM cell line was kept in IMDM medium supplemented with 10% FBS. K562, SEM and MV-4;11 cell lines expressing HLA-A2 were generated by γ-retroviral transduction with pMP71 vector carrying HLA-A2 molecule. K562 cells with FLT3 and THP1 cells overexpressing FLT3 were generated by transducing the pMP71 vector carrying wild type FLT3. FLT3 and HLA-A2 surface expression of cell lines were assessed by FACS staining with antibodies against human FLT3-Alexa Fluor 647 (BD Biosciences, San Jose, CA, USA) and human HLA-A2-PE (Clone BB7.2, BD Biosciences, San Jose, CA, USA). FACS analysis was done with BD FACS Canto II (BD Biosciences, San Jose, CA, USA) and data was analyzed with FlowJo version 10.0.8 (Tree Star, Inc., Ashland, OR, USA). FACS plots are shown in supplementary figure 3.

### Immunization of ABabDII mice

All animal experiments were performed according to institutional and national guidelines and regulations after approval by the responsible authority (Landesamt für Gesundheit und Soziales, Berlin). Predicted peptide was dissolved in appropriate solvent as indicated by the supplier to a concentration of 2mg/ml. Mice were primed on day 0 and immunized on day 21 with 150 µg of peptide in a 1:1 solution of incomplete Freund’s adjuvant (IFA) and 50 µg CpG1826 by subcutaneous injection. Blood was collected 7 days after each boost and blood cells were cultured with 10^-6^ M peptide overnight. Peripheral response was analyzed by intracellular IFN-γ staining of blood cells after overnight culture.

Mice with IFN-γ-secreting CD8^+^ T cells in the periphery were sacrificed. Spleen and inguinal lymph nodes of reactive mice were collected. CD4^+^ T cells were depleted by CD4 microbeads (Miltenyi Biotech, Bergisch Gladbach, Germany). 1x10^6^ splenocytes were seeded per well of a 24-well plate and expanded for 10 days in RPMI 1640 medium supplemented with 10% FBS gold, HEPES, NEAA, Sodium Pyruvate, 50 µM β-mercaptoethanol, 20 IU/ml human IL-2 and 10^-8^ M peptide. Splenocytes were stimulated with 10^-6^ M peptide for 4 h before mouse IFN-γ secretion assay (Miltenyi Biotech, Bergisch Gladbach, Germany). The cells were treated with Fc Block, stained with antibodies against mouse CD3-APC and mouse CD8-PerPC (BD Biosciences, San Jose, CA, USA). IFN-γ secreting CD8^+^ T cells were sorted with BD FACS Aria III (BD Biosciences, San Jose, CA, USA) to RTL lysis buffer for RNA isolation with RNeasy Micro Kit (Qiagen, Hilden, Germany).

### Identification and cloning of TCRs

5’RACE-ready cDNA was synthesized with SMARTer RACE kit (Clontech, CA, USA) according to instructions of the manufacturer. cDNA was diluted 1:3 prior to use. TCRA and TCRB variable chains were amplified by 5’RACE-PCR in a 50 μL reaction mix of 5 μL diluted cDNA, 2X Q5 Hot Start Hihg-Fidelity master mix (New England Biosciences, Ipswich, MA, USA), 5 μL forward primer from the SMARTer RACE kit (10X Universal Primer A Mix (UPM)) and 0.5 μM reverse primers for TCRA: 5’-CGGCCACTTTCAGGAGGAGGATTCGGACC-3’ or TCRB:5’- CCGTAGAACTGGACTTGACAGCGGAAGTGG-3’. Initial denaturation was done at 98°C for 2 min seconds followed by 30 cycles of denaturation at 98°C for 30 s, annealing at 72°c for 30 s and elongation at 72°C for 45 s. Annealing temperature was decreased by 2°C at every 5 cycles for the first 10 cycles. Reaction was carried out for total 35 cycles. Final elongation was done at 72°C for 5 min.

PCR products were separated on 2% gel. Bands corresponding to the correct size were eluted from the gel and cloned using Zero Blunt TOPO PCR Cloning Kit (Invitrogen) and sequenced with SP6 primer. Dominant TCR-α/β chains were selected and paired. The TCR constant regions were replaced with mouse counterparts. Paired TCR-α/β chains were linked with a p2A element. TCR cassette was codon optimized, synthesized by GeneArt (Thermo Fisher Scientific, Waltham, MA, USA) and cloned into pMP71 by restriction site cloning.

**Virus Production**

HEKT-GALV-g/p cells were transfected with 18 µg pMP71 vector carrying the TCR cassette. The virus supernatant was collected 48 h and 72 h after transfection. 1x10^6^ human PBMCs were stimulated on anti-CD3/anti-CD28 coated plates in RPMI 1640 medium supplemented with 10% FBS, HEPES, 100 IU/ml IL-2 and transduced with virus supernatant at 48 h and 72 h after stimulation. Transduction efficiency was determined by FACS staining for human CD8-APC (BD Biosciences, San Jose, CA, USA) and mouse TRBC-PE (Biolegend, San Diego, CA, USA). Mouse TRBC antibody binds to the mouse TCR constant region-β that replaced the human constant region of the TCRs. TCR-transduced-hPBMCs were expanded in T cell medium supplemented with 100 IU/ml IL-2 for 10 days and kept in 10 IU/ml IL-2 supplemented medium for 2 days before the co-culture.

**Functional Assays**

T2 cells were loaded with serial dilutions of peptides at 10^-5^ M to 10^-12^ M for peptide titration experiments. Target cells were selected based on their HLA-A2 and FLT3 expression and labeled with 1 µM CFSE (ab113853, Abcam, Cambridge, UK) prior to seeding. 2x10^4^ target cells and 2x10^4^ TCR-transduced cells were seeded in 200uL final volume in a 96-well format to reach 1:1 effector to target ratio. Cell-free supernatant was collected after overnight incubation for detection of IFN-γ secretion by ELISA. Cells were collected for further analysis and stained with antibodies against human CD137-PE (BD Biosciences, San Jose, CA, USA), human CD8-APC-H7 (BD Biosciences, San Jose, CA, USA), mouse TRBC-APC (Biolegend, San Diego, CA, USA) and run on BD FACSCanto II Flow cytometer. Data was analyzed with FlowJo version 10.0.8.

**FLT3 cloning**

Total RNA was isolated from THP1 cell line with RNeasy mini RNA isolation kit (Qiagen, Hilden, Germany). cDNA was synthesized from 500 ng total RNA with Superscript II (Invitrogen) using random hexamer primers according to manufacturer’s instructions. Full length FLT3 was amplified with the primers F:5’-TATGGCGGCCGCGCCACCATGCCGGCGTT-3’ and R:5’- CAGGCTCAGGTCGAAGATTCGTAA-3’. PCR product was cloned into pMP71 by restriction site cloning.

**Collection of human tissues**

Human autopsy brain samples (n = 3) of anonymized adult individuals were obtained from the Department of Neuropathology, Charité-Universitätsmedizin, Berlin. The brain autopsy was performed following written consent for pathological examination according to the law of Berlin. Inclusion criterion was a routine diagnostic neuropathological examination, which excluded a CNS pathology. The brain samples were used to investigate if FLT3 is expressed in human brain tissue. This procedure was approved by the Charité ethics commission (EA1/040/16) and carried out in accordance with the Declaration of Helsinki.

**FLT3 expression profiling**

TissueScan™ human normal cDNA array and human brain cDNA array were purchased from OriGene. Primers were designed to detect 236 bp amplicon of *FLT3* transcript as F: 5’- CTGAATTGCCAGCCACATTTTG- 3’ and R: 5’-GGAACGCTCTCAGATATGCAG- 3’. PCR was performed in a total reaction volume of 25 µL using 2X Q5 Polymerase Master Mix (New England Biosciences, Ipswich, MA, USA) with 0.5 µM of each primer. Initial denaturation was done at 98°C for 30 s followed by 35 cycles of denaturation at 98°C for 10 s, annealing at 66°C for 30 s and elongation at 72°C for 30 s. Final elongation was done at 72°C for 2 min. *GAPDH* was amplified with primers F: 5’- AGAAGGCTGGGGCTCATTTG-3’ and R: 5’- AGGGGCCATCCACAGTCTTC-3’ as internal control. PCR products were visualized on 2% agarose gel.

Cryosectioned human brain tissues weighing between 30mg and 40mg were used for RNA isolation. Total RNA was isolated using the RNeasy Lipid Tissue mini kit (Qiagen, Hilden, Germany) according to the manufacturer’s instructions. cDNA was synthesized from 500ng total RNA with Superscript IV (Invitrogen) using random hexamer primers. *FLT3* and *GAPDH* amplicons were amplified with the primers listed and cycle conditions stated above.

### Western Blot Analysis

Total protein was extracted from 100 mg to 120 mg pieces of provided brain sections and FLT3 positive cell lines using N-PER neuronal protein extraction reagent (Thermo Fisher Scientific, Waltham, MA, USA) according to manufacturer’s instructions. Total protein was quantified with BCA assay. 100 µg total protein from brain sections and 40 µg total protein from control cell lines were mixed with 4X Laemmli sample buffer (Biorad, Hercules, CA, USA) in 30 µL final volume, cooked 95°C for 5 min. The samples were resolved by SDS polyacrylamide gel electrophoresis (PAGE) using pre-casted polyacrylamide gels (Mini-PROTEAN TGX Precast Gel, 4-20%, Biorad, Hercules, CA, USA), transferred to nitrocellulose membrane, probed with a polyclonal rabbit anti-human FLT3 antibody (1:500 dilution, clone 8F2, Cell Signaling Technology, Danvers, MA, USA) and then with HPR-conjugated goat anti-rabbit IgG (1:2000 dilution, Cell Signaling Technology, Danvers, MA, USA). Bands were visualized by chemiluminescence (ECL-Plus; Amersham Pharmacia Biotech, Little Chalfont, UK) detection using Chemidoc MP Imaging System (Biorad, Hercules, CA, USA). Images were analyzed with Image Lab software (Biorad, Hercules, CA, USA). Membrane was stripped for re-probing with monoclonal mouse anti-human β-actin antibody (1:10000 dilution, Sigma-Aldrich, St. Louis, MO,USA) followed by HRP-conjugated anti-mouse IgG (1:25000 dilution, Sigma-Aldrich, St. Louis, MO,USA) for loading control.

### Immunohistochemical stainings

Immunohistochemical stainings were performed on a VENTANA Benchmark XT automated staining instrument according to the manufacturer's instructions. Formalin-fixed, paraffin-embedded (FFPE) tissue sections (4 μm-thick) were de-paraffinized using EZ prep solution (Ventana Medical Systems, Tucson, AZ, USA) for 30 min at 75 °C. Antigen retrieval was accomplished on the automated stainer using CC1 solution (Ventana Medical Systems, Tucson, AZ, USA) for 60 min at 95 °C. Briefly, the FTL3 antibody (Rabbit polyclonal, dilution 1:50, LS-A7148, Lifespan Biosciences inc., Seattle, WA, USA), was applied and developed using the iVIEW DAB Detection Kit (Ventana Medical Systems, Tucson, AZ, USA). All slides were then counterstained with hematoxylin for 4 min. Omission of primary antibodies as control for nonspecific binding of the secondary antibody resulted in absence of any labeling. To validate the immunohistochemical staining, we used positive control tissues fixed and processed in similar manner to the test sections and known to contain the target molecule, e.g. tonsil.
