# Supplementary material for: Intracellular expression of FLT3 in Purkinje cells: implications for adoptive T-cell therapies
Source: Leukemia. 2019 Jan 3;33(4):1039–43. doi: 10.1038/s41375-018-0330-7 (PMC6484709; doi:10.1038/s41375-018-0330-7)
Supplement: Supplementary file 2 — Supplementary figure 1 [file 41375_2018_330_MOESM2_ESM.pdf]

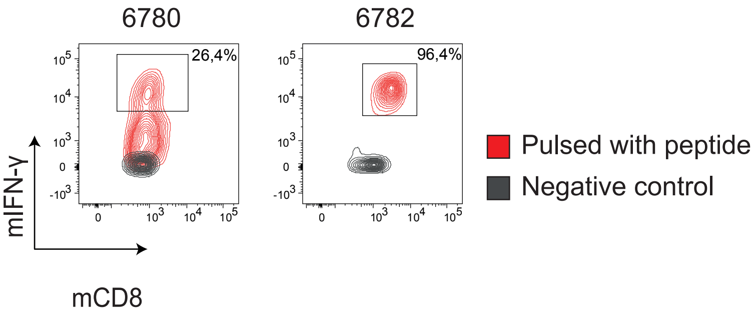

Supplementary figure 1: Mouse IFN-γ-capture assay of splenocytes from mice 6780 and 6782. Splenocytes were expanded in vitro for ten days with 10 nM $FLT3_{982\text{-}991}$ peptide and were pulsed with 1 μM peptide 4 h before the IFN-γ-capture assay. Splenocytes not pulsed with the peptide served as negative control. Populations in the gate are sorted direcly in RTL lysis buffer for RNA Isolation.
