# Supplementary material for: Intracellular expression of FLT3 in Purkinje cells: implications for adoptive T-cell therapies
Source: Leukemia. 2019 Jan 3;33(4):1039–43. doi: 10.1038/s41375-018-0330-7 (PMC6484709; doi:10.1038/s41375-018-0330-7)
Supplement: Supplementary file 3 — Supplementary figure 2 [file 41375_2018_330_MOESM3_ESM.pdf]

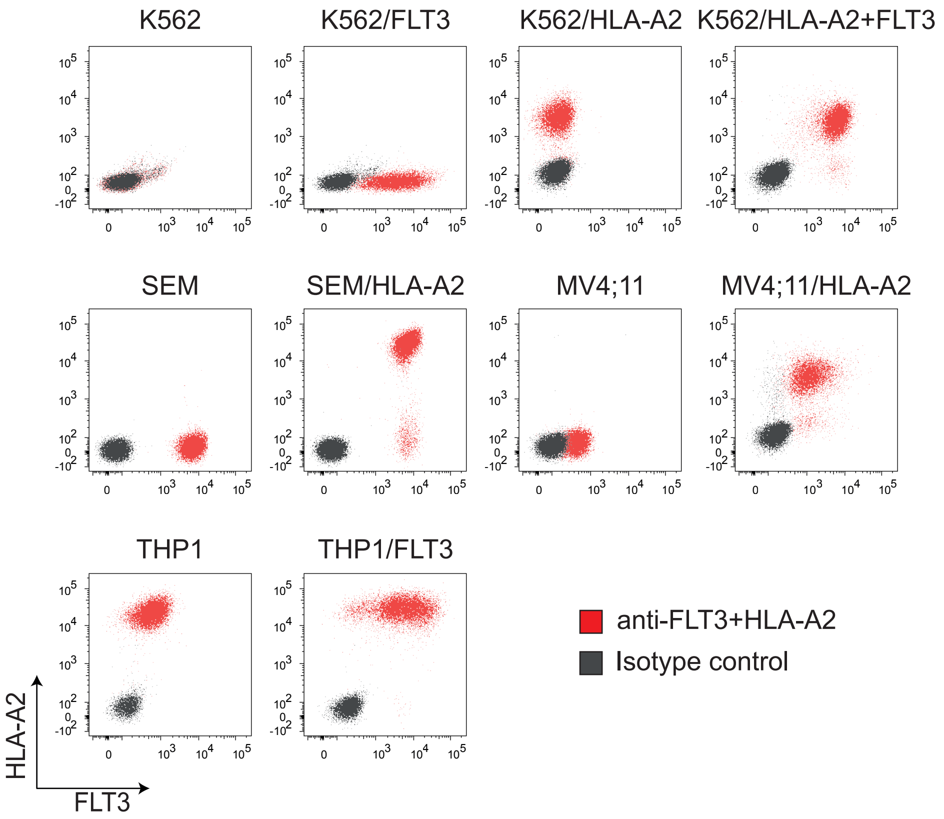

Supplementary figure 2: FACS analysis of cell lines used as targets for expression of FLT3 and HLA-A2 on the surface. Cells were stained with antibodies against human FLT3 conjugated to Alexa Fluor 647 and HLA-A2 conjugated to PE or isotype matched controls. K562, SEM and MV4;11 cell lines were transduced with HLA-A2 molecule. K562, K562/HLA-A2 and THP1 cells were transduced to overexpress FLT3.
