# Supplementary material for: Intracellular expression of FLT3 in Purkinje cells: implications for adoptive T-cell therapies
Source: Leukemia. 2019 Jan 3;33(4):1039–43. doi: 10.1038/s41375-018-0330-7 (PMC6484709; doi:10.1038/s41375-018-0330-7)
Supplement: Supplementary file 4 — Supplementary figure 3 [file 41375_2018_330_MOESM4_ESM.pdf]

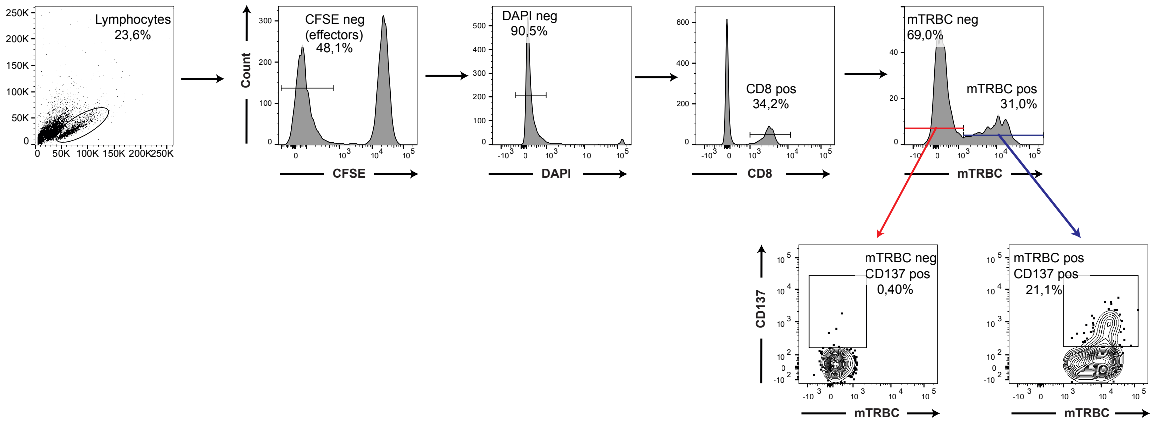

Supplementary figure 3: Gating strategy for the FACS analysis of CD137 expression on TCR-transduced $CD8^{+}$-T cells after overnight co-culture with target cells. Target cells were labeled with 1µM CFSE before seeding to 96-well plate with effector cells. Cells were collected after overnight incubation at 37°C for staining with antibodies against human CD8-APC-H7, human CD137-PE and mouse TRBC-APC. DAPI-positive dead cells were excluded from the analysis.
