# Supplementary material for: Intracellular expression of FLT3 in Purkinje cells: implications for adoptive T-cell therapies
Source: Leukemia. 2019 Jan 3;33(4):1039–43. doi: 10.1038/s41375-018-0330-7 (PMC6484709; doi:10.1038/s41375-018-0330-7)
Supplement: Supplementary file 5 — Supplementary figure 4 [file 41375_2018_330_MOESM5_ESM.pdf]

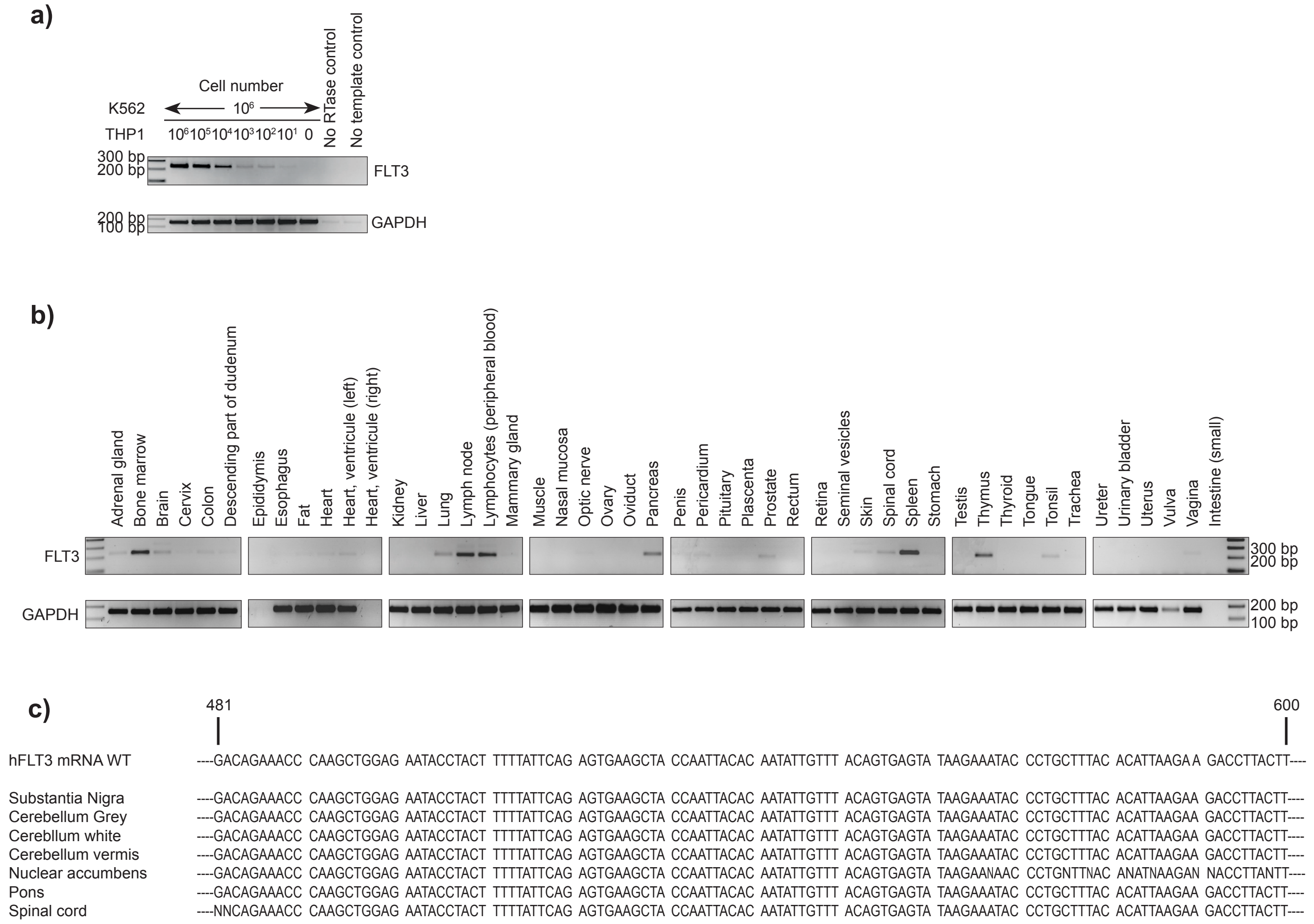

Supplementary figure 4: FLT3 RT-PCR sensitivity and expression profile in healthy human tissues.

a) Serial dilution of FLT3^{pos} THP1 cells were mixed with 10^6 FLT3^{neg} K562 cells and total RNA was isolated. FLT3 RT-PCR was performed with cDNA isolated from each cell dilution. We could detect *FLT3* amplicon after 35 cycles from only 100 FLT3^{pos} cells. As negative controls, PCR mix was set with no cDNA (no template control) and with cDNA mix excluding reverse transcriptase (No RTase control) **b)** *FLT3* amplicon was amplified from commercially available cDNA tissue array representing 48 different healthy human tissues. The transcript was detected in hematopoietic tissues such as bone marrow, lymph node, peripheral blood lymphocytes, spleen, thymus and tonsil. It was also detected in non-hematopoietic tissues such as brain, lung and pancreas. c) Selected PCR products from nerve tissue cDNA array were sent for Sanger sequencing and sequence with the forward PCR primer. 120 bp region of the alingment is shown in the figure. All of the amplicons aligned were to human FLT3 mRNA sequence.
